# Role of food choice motives in the socio-economic disparities in diet diversity and obesity outcomes in Kenya

**DOI:** 10.1371/journal.pone.0302510

**Published:** 2024-05-20

**Authors:** Cecilia Chemeli Maina, Lukas Kornher, Joachim von Braun

**Affiliations:** Department of Economic and Technological Change, Center for Development Research (ZEF), University of Bonn, Bonn, Germany; Independent Consultant, UNITED STATES

## Abstract

**Background:**

The increased prevalence of overweight and obesity, along with high diet diversity, is observed among higher socio-economic groups in Sub-Saharan Africa. One contributing factor to these observed variations is food choice motives. However, the role of these motives in explaining the observed differences has not been thoroughly explored in this context.

**Objective:**

This study investigates whether there are significant differences in food choice motives among socio-economic groups and whether these variations can partly explain the socio-economic disparities in diet diversity and overweight and obesity outcomes.

**Methods:**

This study utilizes cross-sectional data from four counties in Kenya: Kiambu, Murang’a, Uasin Gishu, and Nakuru. The survey employed a three-stage cluster sample design to gather data using structured questionnaires on food choice motives, diet diversity, and anthropometrics from 381 adults in 2022. The mediating effects of 8 food choice motives (health, mood, convenience, sensory appeal, natural content, price, weight control, and familiarity) were analyzed using the Karlson-Holm-Breen method.

**Results:**

The results show that individuals with higher household incomes place greater importance on health, mood, sensory, and weight concerns. The probability of an overweight and obesity outcome increases by 19% for a standard deviation change in the asset score, and by 8% for a standard deviation change in the years of schooling. Sensory motives significantly mediated these relationships. Sensory motives explained 29% of the income-BMI association and 30% of the education-BMI relationship. Higher education was also associated with increased diet diversity (β = 0.36, P < 0.001) mediated by higher health and sensory concerns.

**Conclusions:**

The findings suggest significant differences in food choice motives among socio-economic groups, which contribute to outcomes such as overweight and obesity. Therefore, educational and other policies aimed at reducing obesity should also address food choice motives, while considering the disparities among socio-economic segments within populations.

## Introduction

The rise in the prevalence of overweight and obesity in Sub-Saharan Africa (SSA) is a significant concern because it is associated with an increased risk of non-communicable diseases (NCDs), such as type 2 diabetes, cardiovascular disease, and cancers. The trend in Kenya is like that of other countries in the region, with a steady increase in overweight and obesity prevalence in recent years [1, 2]. According to the Kenya Demographic and Health Surveys (KDHS), the levels of overweight and obesity among women of childbearing age increased from 33% to 45% between 2014 and 2022 [3, 4]. Concurrently, there was an increase from 24% to 31% in the total disability-adjusted life years (DALYs) due to NCDs [5]. Because many countries in SSA have weak healthcare systems, a disproportionate number of deaths have resulted from poor NCD diagnosis, treatment, and control.

The increase in overweight and obesity rates in SSA can be attributed to the adoption of unhealthy diets from globalized food systems; these diets have caused a shift towards highly processed foods that are high in calories, saturated fats, and sugars [6–9]. The rise in social, cultural, and economic interconnectedness has led to the expansion of transnational food companies that promote and distribute these highly processed foods, which are appealing due to their taste, flavour, and texture [10, 11]. This dietary shift has also been driven by several demand-side factors, such as income growth and increased urbanization. As countries experience higher incomes and increased urbanization, there is a corresponding rise in demand for processed and unhealthy foods [7]. For instance, imports of snack foods in Kenya from the global market grew by 20.7% over five years, from 2016 to 2020 [12]. Consequently, this dietary transition—coupled with reduced physical activity—has led to a rise in overweight and obesity rates [7].

Socio-economic position (SEP) factors such as income and education create variations in dietary behaviours and weight outcomes among different socio-economic groups [13]. The 2022 country-wide KDHS data shows that minimum dietary diversity among women aged 20–49 rises with wealth index and education levels. In the lowest wealth quintile, only 22.4% achieve minimum dietary diversity, compared to 65.3% in the highest quintile [4]. Similarly, 19.8% of women with no education achieve minimum dietary diversity, while 65.2% of those with more than a secondary education do. Overweight and obesity levels also increase with wealth and education levels. Among women with no education, 26% are overweight or obese, contrasting with 50% of those with more than a secondary education [4]. In the lowest wealth quintile, approximately one in five women aged 20–49 are overweight or obese, compared to 60% in the highest wealth quintile.

This trend can be attributed to multiple factors within the food system, such as food affordability and accessibility [14]. Individuals with lower incomes tend to spend a larger proportion of their budget on food, which may lead them to purchase cheaper foods that are less diverse and nutritious than those consumed by people with higher incomes [15]. In developed countries, affordable food options often contain high levels of calories, added sugars, and fats, while nutrient-rich foods, such as fresh fruits and vegetables, tend to be more costly [16]. Therefore, individuals of lower socio-economic status consume these readily available foods, resulting in a higher prevalence of overweight and obesity. Developing countries exhibit a reverse pattern, where households with high incomes and education tend to have higher rates of overweight and obesity [17–19]. As both national and personal incomes rise in developing countries, people start having greater access to diverse food choices and the resources to procure them. Consequently, traditional carbohydrate and fiber-rich diets are replaced with new foods that are often higher in fats and sugars and require less time to prepare [7].

Consumer behaviour also plays a key role in the variations seen in food consumption patterns and weight outcomes among different socio-economic groups. Consumer behaviour within the food system encompasses the choices and decisions regarding what food to acquire, store, prepare, cook, and eat [14]. Food choice motives are defined as the reasons and motivations influencing consumers in selecting their food [20]. The food choice process involves decisions that maximize the consumer’s utility. The consumer’s utility function contains a deterministic component related to product attributes and a stochastic component that includes differences in personal preferences that have been shown to differ by socio-economic position [21]. The literature highlights several factors influencing food choices at the individual level. Among these factors, sensory needs play a significant role, encompassing taste, smell, flavor, texture, and overall palatability of food. These sensory aspects have a direct impact on the consumption of certain components like saturated fats, salt, and sugar. These types of food play a crucial role in enhancing the sensory attributes of a diet, making it more flavorful, diverse, and rich [22–24]. Health concerns in food selection refer to the concerns related to disease prevention and overall appearance, which influence the intake of foods perceived to provide these benefits [25, 26]. Negative emotions and overall mood have been demonstrated to influence food choice, particularly when food serves as a coping mechanism [27–29]. Time constraints also play a significant role in food selection decisions, affecting convenience factors such as the time spent on purchasing, preparing, and cooking food [30]. Additionally, familiarity is a key determinant in food selection, it refers to the importance that an individual attaches to eating food they are accustomed to, as opposed to being adventurous and trying new foods [31]. Studies in developed countries have shown that individuals in higher socio-economic positions prioritize health motives when choosing food, while lower SEP groups are more concerned with price, familiarity, and time costs [32–37]. Steptoe et al. using a sample of 358 adults in the UK, found that lower-income groups prioritize price and the familiarity of foods [31]. Robinson et al. utilizing a larger sample of 4,130 adults in the UK and 1,898 adults in the US, found evidence suggesting that individuals from lower socioeconomic backgrounds are less motivated by health when making food choices [33]. This can be attributed to the fact that as income or wages increase, the cost of poor health also rises since being sick means losing time and income [38]. Additionally, individuals in higher SEPs tend to be more knowledgeable about the benefits of healthy diets and have the resources to purchase a balanced diet [32].

The significance of consumer behaviour is often under-researched in developing countries. Despite available research in developed countries, there is still a gap in understanding how food choice motives operate in the context of Sub-Saharan Africa, particularly in Kenya. Therefore, the paper’s main aim is to understand the role of food choice motives in explaining the differences in diet diversity and weight outcomes among different socio-economic groups in Kenya. Studies in SSA have focused on the relationship between SEP and weight outcomes without disentangling the role that psychological factors play. To our knowledge, this is the first study to explore the underlying behavioural pathways through which socio-economic status influences diet diversity and weight outcomes within the Sub-Saharan Africa context, where incomes are rising and obesity is increasingly prevalent among higher socio-economic groups. This analysis will provide valuable insights into the motivations behind individuals in higher SEPs utilizing their increasing incomes to adopt a wider variety of foods, including those that may contribute to higher weight outcomes. Furthermore, it will offer guidance for developing public health interventions that effectively address the escalating prevalence of overweight and obesity in the context of economic growth and evolving dietary behaviours.

This study addresses four research questions: 1) Are there differences in food choice motives by household income and education level? 2) Which food choice motives are associated with diet diversity and weight outcomes? 3) To what extent do food choice motives mediate the relationship between SEP and weight outcomes? 4) To what extent do food choice motives mediate the relationship between SEP and diet diversity? We hypothesize that food choice motives differ by income and education level and significantly contribute to explaining the observed differences in weight outcomes and diet diversity among socio-economic groups. The study successfully achieved its overall aim of understanding the mediating role of food choice motives.

## Materials and methods

### Ethics statement

The study received ethical approval from the University of Bonn and a research license from the Kenya National Commission on Science, Technology, and Innovation (NACOSTI). Additionally, we obtained verbal permission to conduct interviews from the local authorities and written informed consent was obtained from all the participants.

### Inclusivity in global research

Additional information regarding the ethical, cultural, and scientific considerations specific to inclusivity in global research is included in the S1 File).

### Study design

This study uses cross-sectional data collected between 11^th^ May and 18^th^ June 2022 from individuals in four counties in Kenya: Kiambu, Murang’a, Uasin Gishu, and Nakuru counties. These counties were selected based on their varying levels of overweight and obesity rates. According to the 2015 WHO STEPS survey, Murang’a, Kiambu, and Nakuru had the highest rates of overweight and obesity among Kenyan counties, with percentages of 46.5%, 44.6%, and 43.88%, respectively [39]. In comparison, the rate of overweight and obesity in Uasin Gishu County was 24.02%. The high rate of overweight and obesity in the three counties is likely due to high incomes and urbanization, which may contribute to the consumption of unhealthy foods. Nakuru and Kiambu rank among the top five contributors to the country’s economy due to their significant agricultural activities [40].

The sampling strategy employed in this study builds on a prior household survey that was conducted countrywide in 2015 [41]. The sampling frame for the survey was derived from the fifth National Sample Surveys and Evaluation Program (NASSEP V) master sample frame, created by the Kenya National Bureau of Statistics (KNBS), to ensure representation by gender and residence, including both urban and rural areas [41]. The survey used a three-stage cluster sample design, where 200 clusters were selected from one sub-sample of the NASSEP V frame using an equal probability selection method, 30 households were chosen from each cluster using a systematic sampling method with a random start, and one adult individual was randomly selected from all eligible listed household members using a programmed sampling method [41].

The final sample consisted of 381 adults aged 18 and above, with 196 women and 185 men. Among these, 279 were from rural areas, and 102 were from urban areas. This data is representative of the 4 counties where the data was collected. Interviews were conducted in the local language to ensure completeness, using structured questionnaires to gather self-reported information on a range of variables, including demographics (age, gender, household size), diet diversity, food choice motives, and health habits (smoking and alcohol consumption). Additionally, trained enumerators measured weight, height, waist circumference, and hip circumference.

Pregnant women were excluded from analysis involving body mass index (BMI) because physiological changes during pregnancy can affect the results. As a result, 12 participants were removed, reducing the sample size for the BMI analysis to 369.

Power calculations have shown that with a population size of 381 observations and the observed effect sizes from the regressions, using a significance criterion of 95%, the statistical power ranges between 0.85 and 0.98.

### Data

#### Food choice motives

The food choice questionnaire (FCQ) was employed to assess the influences of food selection at the individual level. The original FCQ, developed by Steptoe, Pollard, and Wardle (1995) contained 36 items distributed among 9 factors [31]. Table 1 shows the FCQ structure used in this study which contained 33 items grouped into 8 factors (health, mood, convenience, sensory appeal, natural content, price, weight control, and familiarity). Respondents were asked to rank the importance of each of the motives in their food selection on a typical day (“It is important to me that the food I eat on a typical day…”) using a 7-point Likert scale, that ranged from 1 –Strongly disagree, 2 –Disagree, 3 –Somewhat disagree, 4 –Neither agree nor disagree, 5 –Somewhat agree, 6 –Agree, 7 –Strongly agree [34].

**Table 1:** Food choice questionnaire structure.

It is important to me that the food I eat on a typical day … 1 –Strongly disagree, 2 –Disagree, 3 –Somewhat disagree, 4 –Neither agree nor disagree, 5 –Somewhat agree, 6 –Agree, 7 –Strongly agree
**Factor 1—Health**
Contains a lot of vitamins and minerals
Keeps me healthy
Is nutritious
Is high in protein
Is good for my skin/teeth/hair/nails etc
Is high in fiber and roughage
**Factor 2—Mood**
Helps me cope with stress
Helps me to cope with life
Helps me relax
Keeps me awake/alert
Cheers me up
Makes me feel good
**Factor 3—Convenience**
Is easy to prepare
Can be cooked very simply
Takes very little time to prepare
Can be bought in shops close to where I live or work
Is easily available in shops and supermarkets
**Factor 4—Sensory Appeal**
Smells nice
Looks nice
Has a pleasant texture
Tastes good
**Factor 5—Natural Content**
Contains no additives
Contains natural ingredients
Contains no artificial ingredients
**Factor 6—Price**
Is not expensive
Is cheap
Is good value for money
**Factor 7—Weight Control**
Is low in calories
Helps me control my weight
Is low in fat
**Factor 8—Familiarity**
Is what I usually eat
Is familiar
Is like the food I ate when I was a child

A confirmatory factor analysis was performed to test the hypothesized structures underlying these variables and to establish whether the data fits the original factor structure of the FCQ developed by Steptoe, Pollard, and Wardle (1995) [31]. Several goodness of fit indices indicated acceptability, with the standardized root mean square residual (RMSR) at 0.081, a Tucker—Lewis index (TLI) of 0.781, and a comparative fit index (CFI) of 0.806, suggesting an acceptable fit [42]. Therefore, we proceeded with the analysis using the 8 factors. Factor scores were obtained by summing up the item ratings and then obtaining the average.

#### Diet diversity score

In this study, the household dietary diversity score was used as a measure of diet quality. It is defined as the number of food groups consumed within the past seven days. Developed by the Food and Nutrition Technical Assistance III Project for food security, this indicator is highly correlated with better micronutrient adequacy and diversity in the consumption of micro- and macro-nutrients [43]. Twelve food groups were used to calculate this score. These food groups include cereals, roots and tubers, vegetables, fruits, meat/poultry, eggs, fish and seafood, pulses/legumes/nuts, milk and milk products, oil/fats, sugar/honey, and miscellaneous items. The score is calculated by assigning a value of 1 if a particular food group was consumed, or 0 if it was not. We then sum up the total number of food groups consumed. The score ranges from 0–12, with a higher score indicating high diet diversity.

#### Weight outcomes

The weight outcomes indicator used in this study is the body mass index (BMI) which is widely used to define overweight and obesity. It is obtained by dividing weight by height squared (kg/m^2^). The value was calculated from anthropometric measurements of weight and height collected during the survey from non-pregnant individuals. The BMI scores were grouped into three categories: underweight (<18.5 kg/m^2^), normal (18.5–24.9 kg/m^2^), and overweight and obese (≥25 kg/m^2^) [44]. BMI was categorized to better understand the food choice motives associated with individuals in the overweight and obese categories.

#### Socio-economic measures and covariates

The primary measures of socio-economic position used in this study were the household wealth and the education level of the respondents. Household wealth was assessed using principal component analysis (PCA), which involved creating an asset score based on questions about household asset ownership. This score was determined by considering household ownership of items such as radios, televisions, cars, the type of dwelling, and ownership of agricultural land and livestock. A value of 1 was assigned to indicate household possession of an item, while a value of 0 was assigned if the household did not possess the item. Principal component analysis was then conducted, and the first factor produced was used as the asset score. Additionally, the asset score was used to generate the asset index quintiles, with 1 representing very poor, 2 representing poor, 3 representing average, 4 representing rich, and 5 representing richest. Education, measured in years of schooling, was standardized for easier interpretation of the mediation results.

Covariates used in this analysis were age, gender, household size, marital status, alcohol consumption, smoking status, employment type, and physical activity levels (a binary variable “walk or cycle for more than 10 minutes every day” and time spent sitting daily). These controls were added to account for their potential influence on BMI and diet diversity.

### Statistical methods

All statistical analyses were performed using Stata version 17.0. Collinearity diagnostic tests, including Variance Inflation Factors (VIFs), revealed no evidence of significant collinearity among the food choice variables and also other control variables used in the study. Analysis for the first objective used ordinary least squares (OLS) regression models to assess whether socio-economic measures were associated with food choice motives. We considered each food choice motive as a dependent variable, with education and asset score serving as independent variables. In addition, age, gender, alcohol consumption, and smoking status were included as covariates to account for their potential influence on food choice motives. For the second objective analysis, we used OLS regressions to investigate whether food choice motives, treated as independent variables, were associated with diet diversity. Diet diversity score is a count variable, therefore Poisson regression was additionally conducted for sensitivity analysis which supported our OLS results in terms of the significance of the variables. Ultimately, OLS regression was used in the main analysis and the mediation analysis since the Karlson-Holm-Breen (KHB) outcomes that entail mediation estimation through Poisson regression models are considered experimental [45]. Furthermore, we employed ordered logistic regressions to assess whether food choice motives were linked to BMI categories while controlling for socio-economic variables. The proportional odds assumption was tested using a likelihood ratio test. A non-significant result suggests that there is no significant difference in the coefficients between the models being compared. This implies that the proportional odds assumption holds, meaning that the odds of an outcome remain constant across different outcome levels. Lastly, for the third and fourth objectives, we conducted mediation analyses using only the food choice motives that displayed significant associations with both socio-economic variables and either BMI or diet diversity as mediators.

In this study, the Karlson-Holm-Breen (KHB) method was used to conduct the mediation analysis, which examines whether the effect of a predictor variable *X* on the outcome variable *Y* is partially explained by a third mediator variable *Z* [45]. The effect of the independent variable *X* on the dependent variable *Y*, when there is no mediator, is called the total effect. The total effect can be broken down into two parts: one part that is mediated by *Z* called the indirect effect and another part that is unmediated by *Z* called the direct effect. In this study, there are two main outcome variables: diet diversity, which is treated as a continuous variable, and BMI with three ordered categories. Therefore, both linear and non-linear probability models were used. The total effect is obtained from the reduced model shown in Eq (1) below. This model only contains the predictor variable with no mediator.


$$Y_{i}=\;\alpha_{0}+\alpha_{1}X_{1i}+\alpha_{2}X_{2i}+\epsilon_{i}$$ (1)

In this equation, *Y*_*i*_ is the outcome variable for the individual *i*, *α*_*0*_ is the constant term, *X*_*1i*_ is the predictor variable whose effect is to be decomposed and *α*_*1*_ is the total effect. *X*_*2i*_ is a vector of variables that are determinants of *X*, *Z*, and *Y* and confound the direct and indirect effects. In this analysis, we control for the confounding influence of age, gender, household size, marital status, physical activity, smoking status, alcohol consumption, and employment type. The full model is shown in Eq (2) below.


$$Y_{i}=\;\beta_{0}+\beta_{1}X_{1i}+\beta_{2}X_{2i}+\beta_{3}Z_{i}+\mu_{i}$$ (2)

Here *Z*_*i*_ is included as the variable that is hypothesized to mediate the *X*-*Y* relationship. *β*_*1*_ is the direct effect of *X* on *Y*, given *Z*, *β*_*3*_ is the partial effect of *Z* on *Y*, given *X* and *μ*_*i*_ is the error term. In linear regression models where Y is continuous, the difference in the coefficients for *X*_*1i*_ in the two equations would be the indirect effect (*α*_*1 -*_ *β*_*1*_), which is the magnitude to which the *X-Y* relationship is explained or mediated by *Z*.

In non-linear probability models (NLPM), where *Y* is ordered, the coefficients are scaled relative to the residual standard deviation of the underlying linear model [45]. Adding a mediator variable in the full model, which is correlated with the outcome, results in a decrease in the residual standard deviation. Therefore, when comparing coefficients across models it is difficult to distinguish whether coefficient differences are due to mediation or the rescaling of coefficients across models. The KHB method resolves this by regressing the mediator variable, *Z*, on the variable, *X*, and using the residual *R* in the reduced regression model as shown in Eq (3) below so that the residual standard deviation is the same in the reduced model as in the full model.


$$\;Y_{i}={\tilde{\alpha }}_{0}+{\tilde{\alpha }}_{1}X_{1i}+\tilde{\beta }R+{\tilde{\alpha }}_{2}X_{2i}+\varepsilon_{i}$$ (3)

In NLPM, we use Eq (3) as the reduced model to obtain the indirect effect. The full model in Eq (2) offers no greater predictiveness than the reduced model Eq (3) since *R* and *Z* differ only in the component in *Z* that is correlated with *X* therefore the residuals have the same standard deviation σ^’^_R_ = σ_F_ [45]. With σ^’^_R_ being the standard deviation of the residuals in Eq (3), and σ_F_ the standard deviation of the residuals in Eq (2). Additionally, ${\tilde{\alpha }}_{1}=\alpha_{1}$, therefore, the indirect effect is obtained as the difference between the total effect and the direct effect which is divided by a common scale as shown in Eq (4) below:

$$\frac{{\tilde{\alpha }}_{1}}{\sigma_{R}^{\prime }}-\;\frac{\beta_{1}}{\sigma_{F}}=\;\frac{\alpha_{1}-\;\beta_{1}}{\sigma_{F}}$$ (4)

This mediation analysis does not have a causal interpretation because it does not satisfy the two sequential ignorability assumptions (SIA) [46, 47]. The first assumption is that the predictor variables in this analysis (asset score and education) are independent of unobservables, given the covariates; this assumption can be satisfied with a randomized experiment that was not conducted in this study. The second assumption is that the mediator variables (the food choice factors) are independent of unobserved characteristics (such as cultural influences and genetic predispositions), given the covariates and the predictor variable; this condition was also not satisfied in this setting and it is therefore impossible to rule out self-selection bias. In as much as we try to control for some confounders in this analysis, we cannot rule out the possibility of other unobserved confounders that may affect the predictor, mediator, and outcome relationships. Therefore, the analysis does not claim causal effects.

The food choice motives used in this mediation analysis were those significantly associated with both the socio-economic variables and the outcome variables—either BMI or diet diversity based on objectives 1 and 2, similar to the analytical approach by Robinson et al. (2022) [33]. We conducted the mediation analysis, including multiple mediators whenever possible, to assess the contribution of each mediator. Average partial effects (APE) were reported for the ordered logit model for ease of interpretation on the probability scale. The APE is not constant across ordered outcomes; therefore, this paper shows the decomposition using the average partial effect on the probability of an overweight and obese outcome. For the APE results the confounding ratios and percentages are the same as those of the regression coefficients. The standard errors of difference are not known for the APE method, therefore, we rely on the ordered logit coefficients to establish the significance of the differences. The alpha level that defines statistical significance was 0.10.

## Results

### Sample characteristics and food choice motives

Overweight and obesity rates in the selected counties changed between 2015 and 2022. The current prevalence rates are as follows: Nakuru at 55.8%, Kiambu at 46.7%, Murang’a at 45.6%, and Eldoret at 40.7%. Descriptive statistics of the sample are presented in Table 2. The average BMI for the sample was 25.1 kg/m^2^, which falls in the overweight category. The top food choice motives for the sample population were price, convenience, and health motives while familiarity and weight concerns were ranked the lowest.

**Table 2:** Characteristics of the study participants.

	Mean	SD	IQR
Age (years)	52.40	15.11	23.00
Education (years)	8.70	4.46	5.00
Married (Yes = 1)	0.69	0.46	1.00
Asset score	0.00	0.10	1.90
Household size	2.83	1.49	1.00
BMI (kg/m^2^)	25.07	5.63	7.87
Walk or Cycle more than 10mins daily (= 1 if yes)	0.73	0.45	1.00
Time sitting daily (Minutes)	192.46	135.96	120.00
Person prepares food employed (= 1 if yes)	0.34	0.48	1.00
Diet diversity score	8.48	1.34	1.00
Smoke (= 1 if yes)	1.37	0.69	0.00
Drink alcohol (= 1 if yes)	1.49	0.74	1.00
Food choice motives			
Health	5.57	1.49	1.00
Mood	5.24	1.45	1.50
Sensory	5.31	1.34	2.00
Weight	5.06	1.67	2.00
Convenience	5.70	1.22	1.00
Price	5.84	1.18	1.00
Familiarity	5.01	1.61	1.67
Natural	5.47	1.58	1.667

*** Significant at 1% level, ** Significant at 5% level, * Significant at 10% level

### Association between socioeconomic variables and food choice motives

Regression results with food choice motives as dependent variables showed that measures of SEP were significantly associated with food choice motives (see Tables 3 and 4). An increase of one standard deviation in the asset score was linked to higher ratings for health, mood, sensory, and weight concerns and lower ratings for price concerns.

**Table 3:** Food choice motives by income group.

	Poorest		Second		Middle		Fourth		Richest	
	Mean	SD	Mean	SD	Mean	SD	Mean	SD	Mean	SD
Health	5.18***	1.32	5.52	1.21	5.73	0.94	5.54	1.19	5.87	0.94
Mood	4.70***	1.72	5.21	1.32	5.51	1.15	5.13	1.46	5.64	1.41
Sensory	4.07***	1.32	4.74	1.20	5.86	0.89	5.83	1.06	6.20	0.86
Weight	4.51**	1.83	5.07	1.63	5.25	1.42	5.10	1.69	5.37	1.64
Convenience	5.61**	1.37	6.06	0.83	5.69	1.01	5.66	1.28	5.46	1.45
Price	5.94***	1.21	6.01	1.00	6.19	0.71	5.55	1.36	5.50	1.36
Familiarity	5.26	1.35	5.09	1.46	5.25	1.44	4.82	1.67	4.75	2.03
Natural	5.06*	1.87	5.40	1.45	5.71	1.40	5.61	1.54	5.57	1.55

Mean values were significantly different among income groups (ANOVA). Standard deviation reported in parentheses:

*** Significant at 1% level,

** Significant at 5% level,

* Significant at 10% level

**Table 4:** Linear regression examining the SEP predictors of food choice motives.

	Health	Mood	Sensory	Weight	Convenience	Price	Familiarity	Natural
Years schooling	0.21* (0.12)	0.16 (0.17)	0.09** (0.04)	0.02 (0.20)	-0.09 (0.15)	-0.14 (0.14)	-0.09*** (0.02)	0.09 (0.18)
Asset score	0.05* (0.02)	0.09** (0.04)	0.18*** (0.04)	0.15** (0.07)	-0.07 (0.05)	-0.10** (0.05)	-0.06 (0.05)	0.05 (0.04)
Gender (Male = 1)	-0.06 (0.12)	-0.17 (0.18)	-0.11 (0.16)	0.03 (0.21)	-0.10 (0.16)	0.18 (0.15)	-0.02 (0.21)	-0.16 (0.20)
Age	-0.02*** (0.00)	-0.01* (0.00)	-0.00 (0.00)	-0.01** (0.00)	-0.00 (0.00)	-0.00 (0.00)	-0.02*** (0.01)	0.00 (0.01)
Constant	6.73*** (0.23)	5.90*** (0.34)	5.06*** (0.33)	5.50*** (0.42)	6.18*** (0.29)	6.17*** (0.25)	6.94*** (0.44)	5.70*** (0.34)
R-squared	0.13	0.05	0.12	0.05	0.01	0.03	0.09	0.02

Coefficient estimates are shown with robust standard errors (adjusted for heteroscedasticity) in parentheses.

*** Significant at 1% level,

** Significant at 5% level,

* Significant at 10% level

**Fig 1:**
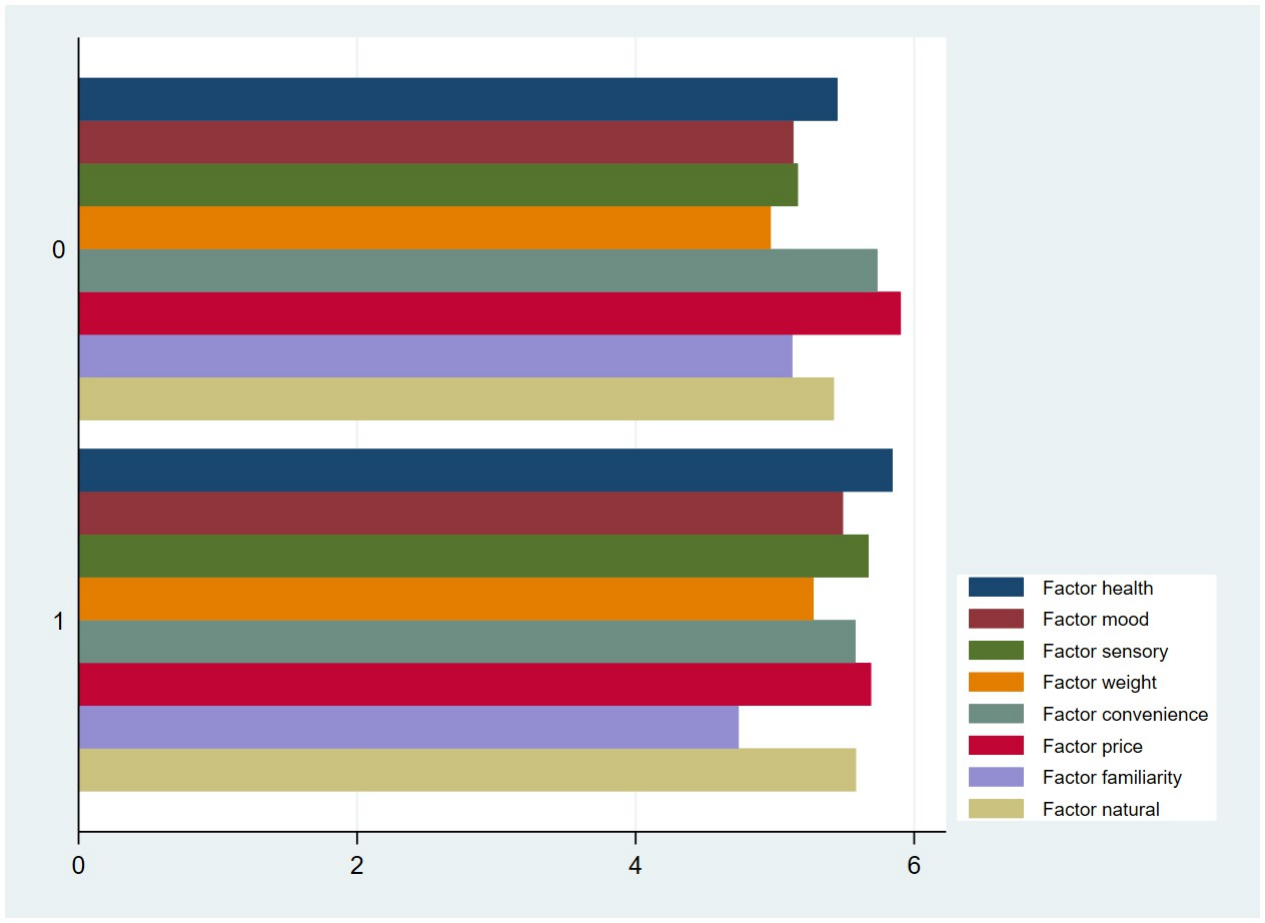
Food choice motives by education levels. Education levels are represented numerically: ’1’ denotes completion of secondary school and beyond, while ’0’ signifies education levels up to incomplete secondary school.

Convenience, familiarity and natural motives were not significantly associated with the asset score. Higher education was associated with being less motivated by familiarity concerns and being more motivated by health and sensory motives when selecting food. Mood, weight, convenience, price, and natural motives were not significantly associated with education levels. Fig 1 shows the relationship between education level and food choice motives. Complete regression tables, including results of other regressors, are shown in S2 File in the Supporting Information.

### Association between food choice motives, diet diversity, and BMI

Various food choice motives were associated with diet diversity as shown in Table 5. Higher concerns for health and sensory aspects were associated with an increase in diet diversity by 0.23 and 0.10, respectively, while higher convenience concerns were associated with a decrease in diet diversity by 0.12. Diet diversity was not significantly associated with other food choice motives. However, mood, weight, price, and natural concerns exhibited a negative relationship, while familiarity displayed a positive association—although these associations were not statistically significant. These results were robust to variations in the model specification, results of the Poisson regression are presented in Table 6. The sign and significance levels of the coefficients are similar to the OLS results.

**Table 5:** Regression results for the estimation of food choice motives associated with diet diversity and BMI.

	Diet Diversity		BMI	
	OLS		Ordered logit	
	Coef.	St. Err.	Coef.	St. Err.
Health	0.23**	0.09	-0.07	0.15
Mood	-0.04	0.07	-0.16	0.12
Sensory	0.10*	0.06	0.58***	0.12
Weight	-0.03	0.05	0.19**	0.09
Convenience	-0.12**	0.06	-0.23**	0.12
Price	0.03	0.07	0.05	0.12
Familiarity	0.07	0.05	0.20**	0.09
Natural	-0.05	0.06	-0.23*	0.12
Constant	6.43***	0.72		
R-squared	0.21			
chi-square			156.13***	
Number of observations	381		369	

Coefficient estimates are shown with robust standard errors.

*** Significant at 1% level,

** Significant at 5% level,

* Significant at 10% level

**Table 6:** Poisson regression results for the estimation of food choice motives associated with diet diversity.

	Coef.	St. Err.
Health	0.027**	0.011
Mood	-0.005	0.008
Sensory	0.012*	0.006
Weight	-0.003	0.006
Convenience	-0.015**	0.007
Price	0.003	0.008
Familiarity	0.009	0.006
Natural	-0.006	0.007
Constant	1.901***	0.079
Pseudo R-squared	0.011	
Number of observations	381	

Coefficient estimates are shown with robust standard errors.

*** Significant at 1% level,

** Significant at 5% level,

* Significant at 10% level

Results indicated that a unit increase in sensory, weight, and familiarity motives would lead to a 0.58, 0.19, and 0.20 increase in the log odds of being in a higher BMI category, respectively. A unit increase in convenience and natural motives would lead to a decrease in the log odds of being in a higher BMI category (results presented in Table 5). The likelihood ratio tests performed to test the proportional odds assumption had a non-significant result which indicated that the relationship between groups is the same. The coefficients for health, mood, and price concerns were not statistically significant although health and mood exhibited a negative relationship with BMI.

### Mediation analyses

The food choice motives used as mediators in the mediation analysis between SEP and BMI were sensory, weight, and familiarity concerns because they were significantly associated with both SEP variables and BMI. In the SEP and diet diversity mediation analysis, health and sensory motives were used as mediators. Other food choice motives that did not exhibit significant associations with either the outcome variables or SEP variables were not used in the mediation analysis.

The KHB estimates of the total, direct, and indirect effects of the socio-economic key variables through food choice motives on BMI and diet diversity are presented in Tables 7 and 8. Table 7 displays the estimated total, direct, and indirect effects of the asset score and years of schooling on BMI. The ordered logit models reveal a positive association between household income and education with BMI, holding other covariates constant. The total effect of the asset score on BMI shows that a one standard deviation increase in the asset score results in a 1.04 increase in the log odds of being in a higher BMI category. The average partial effects indicate that, on average, the probability of an overweight and obese BMI outcome increases by 19 percentage points for a one standard deviation change in the asset score. The relationship between household income and BMI is significantly mediated by sensory and weight concerns. An increase in the asset score leads to higher sensory and weight concerns, resulting in a six-percentage point increase in the probability of being overweight and obese. Sensory concerns account for 28.97% of the household income-BMI association (total effect contribution), while weight concerns explain 3.01% of the household income-BMI association.

**Table 7:** KHB decomposition results of the direct, indirect, and total effects on outcome BMI.

Socio-economic variable	Mediators		Ordered logit		Average Partial Effects for outcome overweight and obese		Indirect effect contribution	Total effect contribution
			Coef.	Std. Err	Coef.	Std. Err		
Asset score	Sensory Weight	Reduced model (total effect)	1.04***	0.18	0.19***	0.03		
		Full model (direct effect)	0.71***	0.20	0.13***	0.04		
		Difference (indirect effect)	0.33***	0.09	0. 06			
		Confounding ratio	1.47					
		Confounding percentage	31.98					
	**Contribution**	Sensory	0.30	0.09	0.06	0.01	90.58	28.97
		Weight	0.03	0.04	0.01	0.01	9.42	3.01
Education	Sensory Familiarity	Reduced model	0.44**	0.14	0.08**	0.03		
		Full model	0.32**	0.15	0.06**	0.03		
		Difference	0.11**	0.05	0.02			
		Confounding ratio	1.34					
		Confounding percentage	25.91					
	**Contribution**	Sensory	0.13	0.04	0.02	0.01	113.85	29.50
		Familiarity	-0.02	0.02	-0.00	0.00	-13.85	-3.59

*** Significant at 1% level,

** Significant at 5% level,

* Significant at 10% level

**Table 8:** KHB decomposition results of the direct, indirect, and total effects on outcome diet diversity score.

Socio-economic variable	Mediators		OLS			
			Coef.	Std. Err	Indirect effect contribution	Total effect contribution
Asset score	Health Sensory	Reduced model (total effect)	0.09	0.07		
		Full model (direct effect)	0.06	0.07		
		Difference (indirect effect)	0.03	0.02		
		Confounding ratio	1.46			
		Confounding percentage	31.78			
	**Contribution**	Health	0.02	0.01	60.85	19.34
		Sensory	0.01	0.01	39.15	12.44
Education	Health Sensory	Reduced model	0.36***	0.08		
		Full model	0.31***	0.08		
		Difference	0.05**	0.02		
		Confounding ratio	1.17			
		Confounding percentage	14.81			
	**Contribution**	Health	0.04	0.02	73.15	10.84
		Sensory	0.01	0.01	26.85	3.98

*** Significant at 1% level,

** Significant at 5% level,

* Significant at 10% level

The mediation models investigating the relationship between years of schooling and BMI revealed a positive and significant association, indicating that education is also linked to overweight and obesity. The average partial effects show an 8% increase in the probability of being overweight and obese for a one standard deviation increase in the years of schooling.

Sensory and familiarity concerns significantly explained this relationship. Higher levels of education are associated with increased sensory concerns and decreased familiarity concerns, resulting in an overall 2% higher likelihood of being overweight and obese. Sensory concerns account for 29.50% of the education-BMI relationship. In contrast, familiarity concerns have a negative indirect effect, explaining -3.59% of the education-BMI relationship.

**Fig 2:**
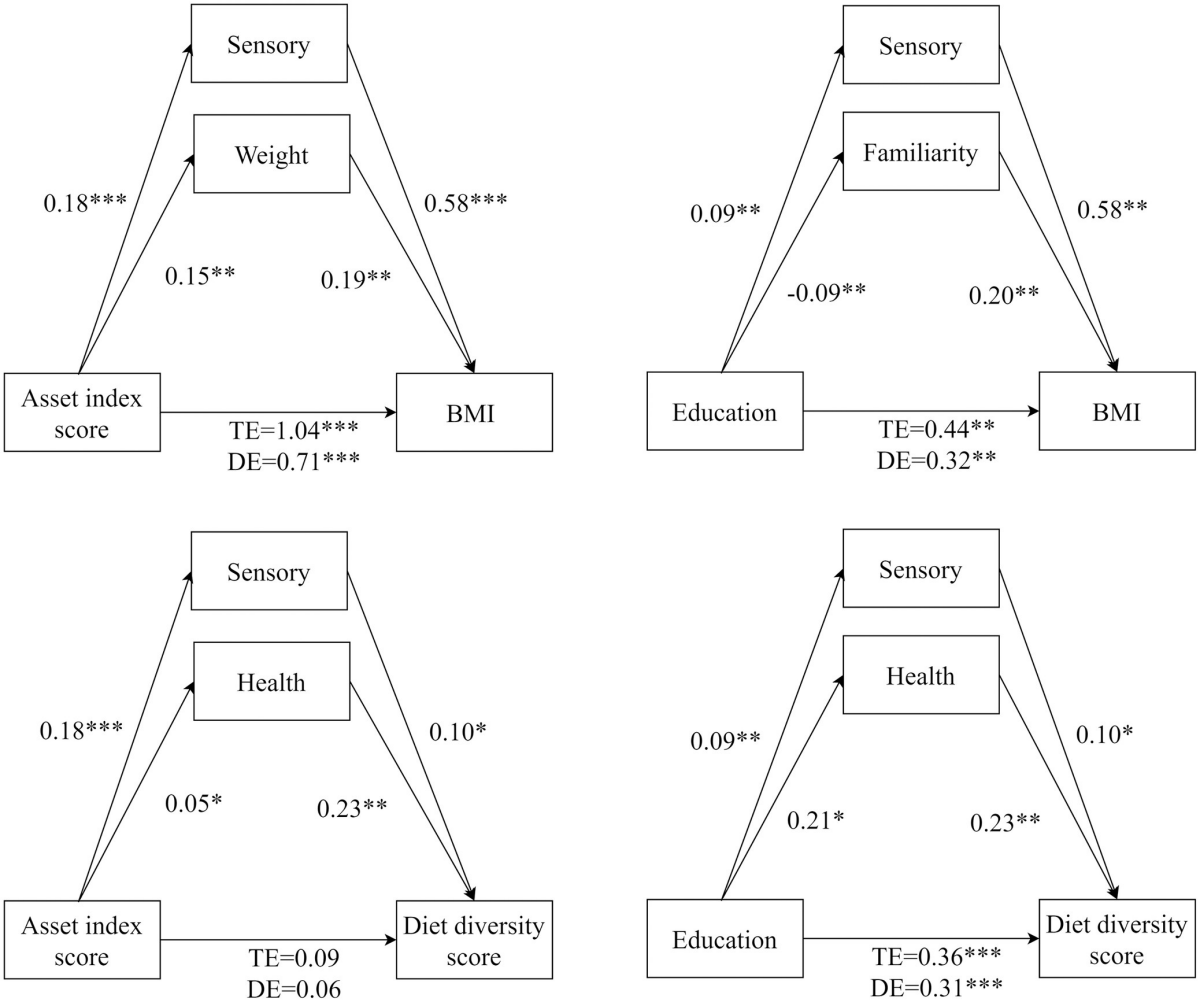
Direct and indirect effect regression coefficients. Mediation models between measures of SEP and diet diversity, BMI. Values represent the individual regression coefficients, TE is the total effect, and DE is the direct effect. *** Significant at 1% level, ** Significant at 5% level, * Significant at 10% level.

The results of the mediation analysis for the diet diversity score revealed that higher education was positively associated with increased diet diversity while controlling for other relevant factors as presented in Table 8. The coefficient representing the total effect was statistically significant (β = 0.36, P < 0.001) for the education-diet diversity score relationship. This relationship was found to be positively mediated by health and sensory concerns, with confounding percentages of 10.84% and 3.98%, respectively. However, health and sensory motives did not significantly mediate the relationship between the asset score and diet diversity, as none of the coefficients in this mediation analysis reached statistical significance. Fig 2 provides a summary of the mediation results with the coefficient estimates from the linear and ordered logit regressions.

## Discussion

The counties included in the study exhibited high rates of overweight and obesity among the sampled participants, surpassing 40%. This high prevalence is of concern for public health, as overweight and obesity serve as risk factors for various non-communicable diseases (NCDs). The study also found significant differences among socio-economic groups in terms of their food consumption motives, dietary behaviours, and weight outcomes. The primary objective was to investigate whether the variations in the importance attributed to food choice motives partly account for the relationship between socio-economic factors and outcomes, such as BMI and diet diversity.

Results from objective 1 revealed that there were differences in the importance placed on food choice motives by socio-economic status. Individuals with higher asset scores rated health, mood, sensory, and weight concerns higher, while rating price concerns lower. These findings suggest that individuals with more resources can consider various food choice concerns without being constrained by price.

Additionally, more years of schooling was correlated with increased concerns placed on health and sensory motives when making food choices, and less concern with familiarity. This indicates that higher education may result in a greater focus on the healthiness of food and a reduced food neophobia [48]. Furthermore, higher health and sensory motives predicted greater diet diversity whereas an emphasis on convenience concerns was linked to lower dietary diversity. A stronger emphasis on sensory, weight, and familiarity motives was associated with a higher likelihood of belonging to a higher BMI category, while an increased focus on convenience and natural motives was associated with a reduced likelihood of being in a high BMI category.

Empirical results from the mediation analysis support previous findings in developing countries, indicating that higher socio-economic status is associated with a greater likelihood of being overweight and obese [49]. The findings reveal that a one-standard-deviation increase in the asset score increases the likelihood of overweight and obesity by 19 percentage points, while a one-standard-deviation increase in years of schooling raises the likelihood by 8 percentage points. Sensory concerns positively and significantly mediated these relationships. Individuals with higher socio-economic status rate sensory concerns highly, which, in turn, is associated with a higher probability of overweight and obesity outcomes. These findings suggest that as household income and education levels increase, households can afford to incorporate a wider variety of foods into their diets while considering sensory preferences. Consequently, as socio-economic status improves, individuals diversify their diets, embracing a variety of foods that cater to their sensory needs that were previously inaccessible due to lower incomes. These food choices, in turn, elevate their risk of overweight and obesity.

These findings also provide support for greater diet diversity among individuals with higher socio-economic status. One standard deviation increase in education is linked to a 0.36 increase in the diet diversity score. This relationship is also significantly mediated by higher sensory concerns. Elevated sensory concerns imply that individuals seek a wider range of foods in their diet, encompassing various sensory properties like textures, flavours, odours, and colours. However, this inclination may put them at risk of increased consumption of unhealthy foods [48].

Another food choice motive that positively mediated the relationship between household income and BMI was the weight motive, which is consistent with findings in the literature. Robinson et. al (2022) found that higher weight control motives were associated with higher BMI, possibly reflecting that individuals who are already overweight tend to be more concerned about their weight when making food choices [22]. A greater desire to lose weight is also counterintuitively associated with weight gain, possibly due to poor weight loss measures [50].

Furthermore, health concerns also mediated the relationship between education and diet diversity. High health concerns led to greater diet diversity, as improved education enhances knowledge about the significance of nutrition and health. A diverse diet is recommended as one aspect of improving health outcomes.

### Study limitations

The first limitation of this study is the use of cross-sectional data; therefore, we cannot establish causality or rule out reverse causality in our analysis. The study can only show the potential mediating effects of food choice factors, which provides useful insights into the effects of psychological factors on consumption and weight outcomes. The second limitation is the use of BMI which simply measures the weight for height without differentiating fat from lean mass which might misclassify a great percentage of people as obese. The percentage of fat and health risk varies by age, gender, and race and cannot be accurately captured by this measure [51]. Alternative measures of adiposity include waist circumference (measures subcutaneous and visceral adipose tissues), adiposity phenotyping, waist-to-hip circumference ratio, waist-to-height ratio, and a body roundness index. Because these measures eventually tend to be highly correlated with BMI in assessing health outcomes, obesity guidelines recommend the continued use of BMI together with other measures of adiposity. The third limitation is the single measure of diet diversity. Future studies should include other measures of diet quality such as the diet quality index and caloric intake to provide more insights into the influence of food choice motives. While diverse diets are associated with micronutrient adequacy, it does not measure other aspects of a diet such as adequacy, moderation, and balance that are linked to the development of obesity and non-communicable diseases [43]. Despite this, the use of the diet diversity score can provide initial insights into the mediating role of food choice motives on diet quality. Additionally, the sampling design was created to ensure representation by gender and residence only. Future studies should base the design on different socio-economic backgrounds for a more comprehensive analysis of the impact of socio-economic disparities on overweight and obesity.

## Conclusions

The findings in this paper indicate that the increasing prevalence of overweight and obesity among high socio-economic groups can be partially explained by the importance that they place on food choice motives, which are correlated with the household’s socio-economic status. It is important to understand food choice motives, diet diversity, and nutritional outcomes among different population groups to implement relevant policies aimed at improving dietary behaviours.

The study results reveal that adverse weight outcomes among individuals in high socio-economic positions can be partially attributed to sensory food choice motives. Highly palatable foods, which are rich in fats, salt, and sugar, are associated with increased energy intake. Findings also reveal that diet quality is not a mere consequence of socio-economic variables, but that food choice motives explain diet diversity patterns. Particularly, health concerns are significantly associated with high diet diversity. Policy implications may include the following: Educational campaigns need to create awareness and focus on providing information on the bad health consequences of food choices based on sensory impulses. The infiltration of multinational companies that seek to exploit individuals’ predisposition for sensory stimulation for profit can be combated through restricted marketing of energy-dense foods. Investments should be made in the development of low-calorie taste compounds in food that sustain the sensory component but have low calories. Public health messages should emphasize increased diet diversity but also include caution on macronutrient moderation so that individuals are aware of daily recommendations on fat, cholesterol, sugar, and salt intake and take measures to stay within the recommended cut-off points.

## Supporting information

S1 File Inclusivity in global research. (DOCX)

S2 File Full regression results. (DOCX)
