# Supplementary material for: Role of food choice motives in the socio-economic disparities in diet diversity and obesity outcomes in Kenya
Source: PLoS One. 2024 May 20;19(5):e0302510. doi: 10.1371/journal.pone.0302510 (PMC11104636; doi:10.1371/journal.pone.0302510)
Supplement: S1 File — (DOCX) [file pone.0302510.s001.docx]

Inclusivity in global research

PLOS’ policy on inclusivity in global research aims to improve transparency in the reporting of research performed outside of researchers’ own country or community and ensures that PLOS publications reporting global research adhere to high standards for research ethics and authorship. Authors of relevant research articles may be asked to complete the questionnaire below, which outlines ethical, cultural, and scientific considerations specific to inclusivity in global research. This questionnaire may be requested when researchers have travelled to a different country to conduct research, if research uses samples collected in another country, research with Indigenous populations or their lands, or if research is on cultural artefacts. Researchers travelling to another country solely to use laboratory equipment will not normally be required to complete the questionnaire. However, the questionnaire can be requested at the journal’s discretion for any submission – if you have been requested to complete this questionnaire by the PLOS journal you submitted to, please do so.

Please complete the questionnaire below and include this as a Supporting Information file with your manuscript. Note that if your paper is accepted for publication, this checklist will be published with your article in the supporting information files. Please ensure that you reference the checklist in the main body of your manuscript. We suggest adding a subsection ‘Inclusivity in global research’ to your Methods section and adding the following sentence: “Additional information regarding the ethical, cultural, and scientific considerations specific to inclusivity in global research is included in the Supporting Information (SX Checklist)”

The questions have been designed to be applicable to a wide range of study types, and there are subsections for both human subjects research and non-human subjects research. If any of the questions are not relevant to your research please mark them as “N/A” as appropriate.

**Ethical considerations, permits and authorship**

*This section is applicable to all research types.*

Provide details as to who granted permissions and/or consent for the study to take place in the Methods section of your manuscript. This should include the names of **all** ethics boards, governmental organizations, community leaders or other bodies that provided approval for the study. If individuals provided approval refer to these people by their role or title but do not list their name(s).

Reported on page number: 6

If there were any deviations from the study protocol after approval was obtained please provide details of these changes in the Methods section of your manuscript.
Did this study involve local collaborators that are residents of the country where the research was conducted or members of the community studied? If you do not have any authors from said communities, please provide an explanation for this below.

Reported on page number: There were no deviations from the study protocol after the approval.

We collaborated with local chiefs, assistant chiefs, village elders, and community leaders whose support was crucial for our study. They granted us permission to conduct interviews within their areas, helped identify key individuals for the study, and assisted in establishing rapport within the community. We were able to discuss some of our research questions and findings with them to get their input, given their deep understanding of the local context, which enhanced the relevance and validity of our research findings. Most of our discussions were verbal, and the key collaborators were not interested in authorship. Nonetheless, their contributions to our understanding of the local context were crucial and proved invaluable for our study.

Everyone listed as an author should meet PLOS’ criteria for authorship and all individuals who meet these criteria should be included in the author byline, rather than the acknowledgements. For further information please see the journal’s Authorship Policy.

**Human subjects research (e.g. health research, medical research, cross-cultural psychology)**

Did you obtain written informed consent from a representative of the local community or region before the research took place? How did you establish who speaks for the community? Details of written informed consent obtained from study participants should be reported separately in the Methods section of your manuscript.

Before the start of the fieldwork, we identified the locations we needed to visit. Kenya has 47 counties, and we selected 4 counties for our research. After obtaining necessary permissions from the Kenya National Commission on Science, Technology, and Innovation (NACOSTI), we obtained the names and contact information of local authorities in the clusters we intended to visit. The authoritative head of each enumeration area or cluster where our participants were located is the chief, reflecting the administrative system structure. Chiefs are the initial contacts for entering communities. We acquired the names and numbers of chiefs from the Kenya National Bureau of Statistics. If chiefs were unavailable, they referred us to their assistants, village elders, or community leaders who possessed deep community knowledge and could assist us. Verbal communication and consent were employed, and we followed protocol by contacting relevant authorities before reaching participants.

How did members of the local community provide input on the aims of the research investigation, its methodology, and its anticipated outcome(s)?

Members of the local community provided invaluable input in the research investigations. They were extremely helpful in terms of methodology; during data collection, they offered advice on the best way to approach participants, which shaped how we utilized our structured questionnaires. Regarding the aims of the research investigation, we consulted with them on what could be driving the increasing prevalence of overweight and obesity. They shared their perspectives regarding the research topic and the expected outcomes, which helped us contextualize the issue and understand the variations in outcomes by region.

When engaging with the local community, how did you ensure that the informed consent documents and other materials could be understood by local stakeholders?

We ensured that the informed consent documents and other materials could be understood by the local stakeholders first by recruiting expert enumerators with significant experience in the field. We then provided proper training to the enumerators, instructing them to request consent and to immediately halt the interview if participants declined consent. Additionally, we practiced building rapport and approaching participants in a sensitive and appropriate manner. We conducted practice sessions using the main languages of the regions. If participants did not understand English, enumerators used Kiswahili. If participants faced challenges with Kiswahili, we reassigned them to an enumerator who could speak the local language to ensure comprehension of the informed consent and other materials.

Will the findings of the research be made available in an understandable format to stakeholders in the community where the study was conducted (e.g. via a presentation, summary report, copies of publications, etc.)? Please provide details of how this will be achieved.

While we deeply appreciate the importance of sharing research findings with the community stakeholders, at this stage, we do not have specific plans for disseminating the results in the formats you mentioned. However, we understand the significance of making the findings accessible and understandable to those involved. We are still exploring various avenues for effectively communicating the results to the community. This may include organizing presentations, preparing summary reports, and offering copies of publications, among other methods. We are committed to ensuring that the findings are disseminated in a manner that fosters understanding and engagement among community stakeholders.

**Non-human subjects research using specimens/ animals collected as part of the study, or those housed in archival collections. Examples include archaeology, paleontology, botany and zoology.**

Did the permission you obtained from a local authority to perform the study include an agreement on access to outputs and benefit sharing? This may include procedures to enable fair distribution of the benefits and resources arising from the research performed. Please include any details of Prior Informed Consent and Benefit Sharing Agreements obtained. These may be required by field-specific regulations, for example the Convention on Biological Diversity (CBD) and the associated Nagoya Protocol.

N/A

If the material used in your study was imported, please A) provide the year it was imported and B) indicate whether permits were obtained to import/export the materials used, C) provide details of any permits obtained. If this information is not available, please indicate this.

N/A

If you used archival specimens, please state how the material used in your study was acquired by the institute it is held in and provide details of any permits obtained for the original excavations/ sample collection. If this information is not available, please indicate this.

N/A

How was the potential cultural significance of the materials collected in your study to local communities considered in your research design? Were Indigenous peoples and/or local researchers and institutions involved with archaeological excavations / collection of specimens? If so, please provide a description of their involvement.

N/A

If your manuscript includes photographs of human remains please indicate whether authors obtained permission from descendants or affiliated cultural communities to do so.

N/A
