# Supplementary material for: Role of food choice motives in the socio-economic disparities in diet diversity and obesity outcomes in Kenya
Source: PLoS One. 2024 May 20;19(5):e0302510. doi: 10.1371/journal.pone.0302510 (PMC11104636; doi:10.1371/journal.pone.0302510)
Supplement: S2 File — (DOCX) [file pone.0302510.s002.docx]

|  |
| --- |

**SEP predictors of food choice motives**

| Factor Health | Coef. | | St.Err. | t-value | | p-value | [95% Conf | | Interval] | | Sig |
| --- | --- | --- | --- | --- | --- | --- | --- | --- | --- | --- | --- |
| Gender | 0 | | . | . | | . | . | | . | |  |
| Male | -.062 | | .123 | -0.51 | | .612 | -.303 | | .179 | |  |
| Age years | -.015 | | .004 | -3.66 | | 0 | -.023 | | -.007 | | *** |
| Education | 0 | | . | . | | . | . | | . | |  |
| Post secondary | .205 | | .118 | 1.74 | | .082 | -.026 | | .437 | | * |
| Asset score | .046 | | .024 | 1.89 | | .059 | -.002 | | .093 | | * |
| Drinks alcohol | .063 | | .102 | 0.62 | | .538 | -.138 | | .264 | |  |
| Smokes cigarettes | -.365 | | .125 | -2.91 | | .004 | -.612 | | -.119 | | *** |
| Constant | 6.731 | | .228 | 29.57 | | 0 | 6.283 | | 7.178 | | *** |
|  | | | | | | | | | | | |
| Mean dependent var | | 5.569 | | | SD dependent var | | | 1.150 | |  |  |
| R-squared | | 0.128 | | | Number of obs | | | 380 | |  |  |
| F-test | | 8.902 | | | Prob > F | | | 0.000 | |  |  |
| Akaike crit. (AIC) | | 1145.794 | | | Bayesian crit. (BIC) | | | 1173.376 | |  |  |
| **** p<.01, ** p<.05, * p<.1*   \| Factor Sensory \| Coef. \| \| St.Err. \| t-value \| \| p-value \| [95% Conf \| \| Interval] \| \| Sig \| \| --- \| --- \| --- \| --- \| --- \| --- \| --- \| --- \| --- \| --- \| --- \| --- \| \| Gender \| 0 \| \| . \| . \| \| . \| . \| \| . \| \|  \| \| Male \| -.109 \| \| .156 \| -0.70 \| \| .482 \| -.416 \| \| .197 \| \|  \| \| Age years \| .001 \| \| .004 \| 0.33 \| \| .742 \| -.007 \| \| .01 \| \|  \| \| Education \| .092 \| \| .044 \| 2.10 \| \| .037 \| .006 \| \| .178 \| \| ** \| \| Asset score \| .178 \| \| .041 \| 4.38 \| \| 0 \| .098 \| \| .258 \| \| *** \| \| Drinks alcohol \| .128 \| \| .117 \| 1.09 \| \| .275 \| -.102 \| \| .359 \| \|  \| \| Smokes cigarettes \| -.203 \| \| .121 \| -1.68 \| \| .093 \| -.44 \| \| .034 \| \| * \| \| Constant \| 5.06 \| \| .329 \| 15.38 \| \| 0 \| 4.412 \| \| 5.707 \| \| *** \| \|  \| \| \| \| \| \| \| \| \| \| \| \| \| Mean dependent var \| \| 5.315 \| \| \| SD dependent var \| \| \| 1.338 \| \| \| R-squared \| \| 0.119 \| \| \| Number of obs \| \| \| 380 \| \| \| F-test \| \| 6.694 \| \| \| Prob > F \| \| \| 0.000 \| \| \| Akaike crit. (AIC) \| \| 1264.390 \| \| \| Bayesian crit. (BIC) \| \| \| 1291.971 \| \| \| **** p<.01, ** p<.05, * p<.1* \| \| \| \| \| \| \| \| \| \| \| \| \|  \| \| \| \| \| \| \| \| \| \| \| \| | | | | | | | | | | | |
| \| Factor Mood \| Coef. \| \| St.Err. \| t-value \| \| p-value \| [95% Conf \| \| Interval] \| \| Sig \| \| --- \| --- \| --- \| --- \| --- \| --- \| --- \| --- \| --- \| --- \| --- \| --- \| \| Education \| 0 \| \| . \| . \| \| . \| . \| \| . \| \|  \| \| Post secondary \| .165 \| \| .175 \| 0.94 \| \| .346 \| -.178 \| \| .508 \| \|  \| \| Asset score \| .088 \| \| .039 \| 2.28 \| \| .023 \| .012 \| \| .164 \| \| ** \| \| Gender \| 0 \| \| . \| . \| \| . \| . \| \| . \| \|  \| \| Male \| -.18 \| \| .182 \| -0.99 \| \| .325 \| -.538 \| \| .179 \| \|  \| \| Age years \| -.009 \| \| .005 \| -1.68 \| \| .093 \| -.02 \| \| .002 \| \| * \| \| Drinks alcohol \| .127 \| \| .13 \| 0.98 \| \| .329 \| -.129 \| \| .383 \| \|  \| \| Smokes cigarettes \| -.251 \| \| .143 \| -1.76 \| \| .08 \| -.531 \| \| .03 \| \| * \| \| Constant \| 5.903 \| \| .342 \| 17.25 \| \| 0 \| 5.23 \| \| 6.576 \| \| *** \| \|  \| \| \| \| \| \| \| \| \| \| \| \| \| Mean dependent var \| \| 5.237 \| \| \| SD dependent var \| \| \| 1.456 \| \| \| R-squared \| \| 0.055 \| \| \| Number of obs \| \| \| 380 \| \| \| F-test \| \| 3.449 \| \| \| Prob > F \| \| \| 0.003 \| \| \| Akaike crit. (AIC) \| \| 1355.230 \| \| \| Bayesian crit. (BIC) \| \| \| 1382.811 \| \| \| **** p<.01, ** p<.05, * p<.1* \| \| \| \| \| \| \| \| \| \| \| \| \|  \| \| \| \| \| \| \| \| \| \| \| \|  \| Factor weight \| Coef. \| \| St.Err. \| t-value \| \| p-value \| [95% Conf \| \| Interval] \| \| Sig \| \| --- \| --- \| --- \| --- \| --- \| --- \| --- \| --- \| --- \| --- \| --- \| --- \| \| Education \| 0 \| \| . \| . \| \| . \| . \| \| . \| \|  \| \| Post secondary \| .018 \| \| .205 \| 0.09 \| \| .93 \| -.385 \| \| .421 \| \|  \| \| Asset score \| .148 \| \| .067 \| 2.21 \| \| .028 \| .016 \| \| .28 \| \| ** \| \| Gender \| 0 \| \| . \| . \| \| . \| . \| \| . \| \|  \| \| Male \| .034 \| \| .208 \| 0.16 \| \| .872 \| -.375 \| \| .442 \| \|  \| \| Age years \| -.014 \| \| .006 \| -2.21 \| \| .028 \| -.026 \| \| -.002 \| \| ** \| \| Drinks alcohol \| .099 \| \| .15 \| 0.66 \| \| .51 \| -.195 \| \| .393 \| \|  \| \| Smokes cigarettes \| -.244 \| \| .161 \| -1.51 \| \| .131 \| -.561 \| \| .073 \| \|  \| \| Constant \| 5.5 \| \| .418 \| 13.16 \| \| 0 \| 4.678 \| \| 6.322 \| \| *** \| \|  \| \| \| \| \| \| \| \| \| \| \| \| \| Mean dependent var \| \| 5.059 \| \| \| SD dependent var \| \| \| 1.669 \| \| \| R-squared \| \| 0.046 \| \| \| Number of obs \| \| \| 380 \| \| \| F-test \| \| 2.942 \| \| \| Prob > F \| \| \| 0.008 \| \| \| Akaike crit. (AIC) \| \| 1462.590 \| \| \| Bayesian crit. (BIC) \| \| \| 1490.171 \| \| \| **** p<.01, ** p<.05, * p<.1* \| \| \| \| \| \| \| \| \| \| \| \| \|  \| \| \| \| \| \| \| \| \| \| \| \|  \| Factor convenience \| Coef. \| \| St.Err. \| t-value \| \| p-value \| [95% Conf \| \| Interval] \| \| Sig \| \| --- \| --- \| --- \| --- \| --- \| --- \| --- \| --- \| --- \| --- \| --- \| --- \| \| Education \| 0 \| \| . \| . \| \| . \| . \| \| . \| \|  \| \| Post secondary \| -.09 \| \| .153 \| -0.59 \| \| .555 \| -.392 \| \| .211 \| \|  \| \| Asset score \| -.067 \| \| .05 \| -1.33 \| \| .185 \| -.166 \| \| .032 \| \|  \| \| Gender \| 0 \| \| . \| . \| \| . \| . \| \| . \| \|  \| \| Male \| -.105 \| \| .163 \| -0.64 \| \| .523 \| -.426 \| \| .217 \| \|  \| \| Age years \| -.004 \| \| .004 \| -0.96 \| \| .337 \| -.013 \| \| .004 \| \|  \| \| Drinks alcohol \| .074 \| \| .1 \| 0.75 \| \| .455 \| -.121 \| \| .27 \| \|  \| \| Smokes cigarettes \| -.071 \| \| .105 \| -0.68 \| \| .498 \| -.278 \| \| .135 \| \|  \| \| Constant \| 6.18 \| \| .294 \| 20.99 \| \| 0 \| 5.601 \| \| 6.759 \| \| *** \| \|  \| \| \| \| \| \| \| \| \| \| \| \| \| Mean dependent var \| \| 5.695 \| \| \| SD dependent var \| \| \| 1.221 \| \| \| R-squared \| \| 0.014 \| \| \| Number of obs \| \| \| 380 \| \| \| F-test \| \| 0.855 \| \| \| Prob > F \| \| \| 0.529 \| \| \| Akaike crit. (AIC) \| \| 1237.679 \| \| \| Bayesian crit. (BIC) \| \| \| 1265.260 \| \| \| **** p<.01, ** p<.05, * p<.1* \| \| \| \| \| \| \| \| \| \| \| \| \|  \| \| \| \| \| \| \| \| \| \| \| \|  \| Factor Price \| Coef. \| \| St.Err. \| t-value \| \| p-value \| [95% Conf \| \| Interval] \| \| Sig \| \| --- \| --- \| --- \| --- \| --- \| --- \| --- \| --- \| --- \| --- \| --- \| --- \| \| Education \| 0 \| \| . \| . \| \| . \| . \| \| . \| \|  \| \| Post secondary \| -.141 \| \| .144 \| -0.98 \| \| .325 \| -.424 \| \| .141 \| \|  \| \| Asset score \| -.096 \| \| .045 \| -2.13 \| \| .034 \| -.185 \| \| -.007 \| \| ** \| \| Gender \| 0 \| \| . \| . \| \| . \| . \| \| . \| \|  \| \| Male \| .179 \| \| .152 \| 1.18 \| \| .239 \| -.119 \| \| .478 \| \|  \| \| Age years \| -.003 \| \| .004 \| -0.74 \| \| .458 \| -.012 \| \| .005 \| \|  \| \| Drinks alcohol \| .005 \| \| .093 \| 0.05 \| \| .959 \| -.178 \| \| .188 \| \|  \| \| Smokes cigarettes \| -.155 \| \| .095 \| -1.62 \| \| .106 \| -.342 \| \| .033 \| \|  \| \| Constant \| 6.175 \| \| .247 \| 25.02 \| \| 0 \| 5.689 \| \| 6.66 \| \| *** \| \|  \| \| \| \| \| \| \| \| \| \| \| \| \| Mean dependent var \| \| 5.839 \| \| \| SD dependent var \| \| \| 1.179 \| \| \| R-squared \| \| 0.037 \| \| \| Number of obs \| \| \| 380 \| \| \| F-test \| \| 1.716 \| \| \| Prob > F \| \| \| 0.116 \| \| \| Akaike crit. (AIC) \| \| 1202.325 \| \| \| Bayesian crit. (BIC) \| \| \| 1229.906 \| \| \| **** p<.01, ** p<.05, * p<.1* \| \| \| \| \| \| \| \| \| \| \| \| \|  \| \| \| \| \| \| \| \| \| \| \| \|  \| Factor familiarity \| Coef. \| \| St.Err. \| t-value \| \| p-value \| [95% Conf \| \| Interval] \| \| Sig \| \| --- \| --- \| --- \| --- \| --- \| --- \| --- \| --- \| --- \| --- \| --- \| --- \| \| Education \| -.09 \| \| .024 \| -3.78 \| \| 0 \| -.136 \| \| -.043 \| \| *** \| \| Asset score \| -.062 \| \| .047 \| -1.32 \| \| .187 \| -.154 \| \| .03 \| \|  \| \| Gender \| 0 \| \| . \| . \| \| . \| . \| \| . \| \|  \| \| Male \| -.019 \| \| .208 \| -0.09 \| \| .929 \| -.428 \| \| .391 \| \|  \| \| Age years \| -.018 \| \| .006 \| -3.14 \| \| .002 \| -.029 \| \| -.007 \| \| *** \| \| Drinks alcohol \| .27 \| \| .135 \| 2.00 \| \| .047 \| .004 \| \| .536 \| \| ** \| \| Smokes cigarettes \| -.417 \| \| .14 \| -2.97 \| \| .003 \| -.693 \| \| -.141 \| \| *** \| \| Constant \| 6.944 \| \| .441 \| 15.74 \| \| 0 \| 6.077 \| \| 7.812 \| \| *** \| \|  \| \| \| \| \| \| \| \| \| \| \| \| \| Mean dependent var \| \| 5.038 \| \| \| SD dependent var \| \| \| 1.612 \| \| \| R-squared \| \| 0.093 \| \| \| Number of obs \| \| \| 380 \| \| \| F-test \| \| 5.748 \| \| \| Prob > F \| \| \| 0.000 \| \| \| Akaike crit. (AIC) \| \| 1417.349 \| \| \| Bayesian crit. (BIC) \| \| \| 1444.930 \| \| \| **** p<.01, ** p<.05, * p<.1* \| \| \| \| \| \| \| \| \| \| \| \| \|  \| \| \| \| \| \| \| \| \| \| \| \|  \| Factor Natural \| Coef. \| \| St.Err. \| t-value \| \| p-value \| [95% Conf \| \| Interval] \| \| Sig \| \| --- \| --- \| --- \| --- \| --- \| --- \| --- \| --- \| --- \| --- \| --- \| --- \| \| Education \| 0 \| \| . \| . \| \| . \| . \| \| . \| \|  \| \| Post secondary \| .086 \| \| .181 \| 0.48 \| \| .634 \| -.269 \| \| .441 \| \|  \| \| Asset score \| .055 \| \| .041 \| 1.33 \| \| .183 \| -.026 \| \| .136 \| \|  \| \| Gender \| 0 \| \| . \| . \| \| . \| . \| \| . \| \|  \| \| Male \| -.161 \| \| .204 \| -0.79 \| \| .43 \| -.562 \| \| .24 \| \|  \| \| Age years \| 0 \| \| .006 \| 0.04 \| \| .969 \| -.011 \| \| .011 \| \|  \| \| Drinks alcohol \| .019 \| \| .152 \| 0.13 \| \| .899 \| -.28 \| \| .319 \| \|  \| \| Smokes cigarettes \| -.157 \| \| .168 \| -0.94 \| \| .35 \| -.486 \| \| .172 \| \|  \| \| Constant \| 5.697 \| \| .348 \| 16.39 \| \| 0 \| 5.013 \| \| 6.381 \| \| *** \| \|  \| \| \| \| \| \| \| \| \| \| \| \| \| Mean dependent var \| \| 5.469 \| \| \| SD dependent var \| \| \| 1.583 \| \| \| R-squared \| \| 0.016 \| \| \| Number of obs \| \| \| 380 \| \| \| F-test \| \| 1.088 \| \| \| Prob > F \| \| \| 0.369 \| \| \| Akaike crit. (AIC) \| \| 1434.588 \| \| \| Bayesian crit. (BIC) \| \| \| 1462.169 \| \| \| **** p<.01, ** p<.05, * p<.1* \| \| \| \| \| \| \| \| \| \| \| \| \|  \| \| \| \| \| \| \| \| \| \| \| \|   **Box-cox power transformed dependent variable results**   \| bchealth \| Coef. \| \| St.Err. \| t-value \| \| p-value \| [95% Conf \| \| Interval] \| \| Sig \| \| --- \| --- \| --- \| --- \| --- \| --- \| --- \| --- \| --- \| --- \| --- \| --- \| \| Gender \| 0 \| \| . \| . \| \| . \| . \| \| . \| \|  \| \| Male \| -18.682 \| \| 16.468 \| -1.13 \| \| .257 \| -51.064 \| \| 13.701 \| \|  \| \| Age years \| -1.549 \| \| .476 \| -3.25 \| \| .001 \| -2.486 \| \| -.612 \| \| *** \| \| Education \| 0 \| \| . \| . \| \| . \| . \| \| . \| \|  \| \| Post secondary \| 30.418 \| \| 15.713 \| 1.94 \| \| .054 \| -.478 \| \| 61.315 \| \| * \| \| Asset score \| 7.365 \| \| 3.688 \| 2.00 \| \| .047 \| .114 \| \| 14.617 \| \| ** \| \| Drinks alcohol \| .587 \| \| 11.112 \| 0.05 \| \| .958 \| -21.263 \| \| 22.437 \| \|  \| \| Smokes cigarettes \| -37.695 \| \| 12.485 \| -3.02 \| \| .003 \| -62.244 \| \| -13.146 \| \| *** \| \| Constant \| 401.481 \| \| 28.389 \| 14.14 \| \| 0 \| 345.657 \| \| 457.304 \| \| *** \| \|  \| \| \| \| \| \| \| \| \| \| \| \| \| Mean dependent var \| \| 269.352 \| \| \| SD dependent var \| \| \| 142.099 \| \| \| R-squared \| \| 0.128 \| \| \| Number of obs \| \| \| 380 \| \| \| F-test \| \| 11.010 \| \| \| Prob > F \| \| \| 0.000 \| \| \| Akaike crit. (AIC) \| \| 4806.498 \| \| \| Bayesian crit. (BIC) \| \| \| 4834.079 \| \| \| **** p<.01, ** p<.05, * p<.1* \| \| \| \| \| \| \| \| \| \| \| \| \|  \| \| \| \| \| \| \| \| \| \| \| \|  \| bcsensory \| \| Coef. \| \| \| \| St.Err. \| \| \| t-value \| \| \| p-value \| \| [95% Conf \| \| \| \| Interval] \| \| \| \| Sig \| \| \| --- \| --- \| --- \| --- \| --- \| --- \| --- \| --- \| --- \| --- \| --- \| --- \| --- \| --- \| --- \| --- \| --- \| --- \| --- \| --- \| --- \| --- \| --- \| --- \| \| Gender \| \| 0 \| \| \| \| . \| \| \| . \| \| \| . \| \| . \| \| \| \| . \| \| \| \|  \| \| \| Male \| \| -1.706 \| \| \| \| 3.333 \| \| \| -0.51 \| \| \| .609 \| \| -8.26 \| \| \| \| 4.847 \| \| \| \|  \| \| \| Age years \| \| .067 \| \| \| \| .093 \| \| \| 0.73 \| \| \| .468 \| \| -.115 \| \| \| \| .25 \| \| \| \|  \| \| \| Education \| \| 1.681 \| \| \| \| .956 \| \| \| 1.76 \| \| \| .079 \| \| -.198 \| \| \| \| 3.56 \| \| \| \| * \| \| \| Asset score \| \| 3.879 \| \| \| \| .874 \| \| \| 4.44 \| \| \| 0 \| \| 2.159 \| \| \| \| 5.598 \| \| \| \| *** \| \| \| Drinks alcohol \| \| 3.167 \| \| \| \| 2.48 \| \| \| 1.28 \| \| \| .202 \| \| -1.709 \| \| \| \| 8.044 \| \| \| \|  \| \| \| Smokes cigarettes \| \| -5.508 \| \| \| \| 2.388 \| \| \| -2.31 \| \| \| .022 \| \| -10.204 \| \| \| \| -.812 \| \| \| \| ** \| \| \| Constant \| \| 43.956 \| \| \| \| 7.22 \| \| \| 6.09 \| \| \| 0 \| \| 29.759 \| \| \| \| 58.152 \| \| \| \| *** \| \| \|  \| \| \| \| \| \| \| \| \| \| \| \| \| \| \| \| \| \| \| \| \| \| \| \| \| Mean dependent var \| \| \| \| 49.734 \| \| \| \| \| \| SD dependent var \| \| \| \| \| \| 28.374 \| \| \| \| \| \| R-squared \| \| \| \| 0.122 \| \| \| \| \| \| Number of obs \| \| \| \| \| \| 380 \| \| \| \| \| \| F-test \| \| \| \| 6.913 \| \| \| \| \| \| Prob > F \| \| \| \| \| \| 0.000 \| \| \| \| \| \| Akaike crit. (AIC) \| \| \| \| 3584.583 \| \| \| \| \| \| Bayesian crit. (BIC) \| \| \| \| \| \| 3612.164 \| \| \| \| \| \| **** p<.01, ** p<.05, * p<.1* \| \| \| \| \| \| \| \| \| \| \| \| \| \| \| \| \| \| \| \| \| \| \| \| \|  \| \| \| \| \| \| \| \| \| \| \| \| \| \| \| \| \| \| \| \| \| \| \| \| \| bcweight \| \| Coef. \| \| \| \| St.Err. \| \| t-value \| \| \| \| p-value \| \| [95% Conf \| \| \| \| Interval] \| \| \| Sig \| \| \| \| Gender \| \| 0 \| \| \| \| . \| \| . \| \| \| \| . \| \| . \| \| \| \| . \| \| \|  \| \| \| \| Male \| \| .802 \| \| \| \| 5.778 \| \| 0.14 \| \| \| \| .89 \| \| -10.559 \| \| \| \| 12.164 \| \| \|  \| \| \| \| Age years \| \| -.241 \| \| \| \| .166 \| \| -1.45 \| \| \| \| .148 \| \| -.567 \| \| \| \| .086 \| \| \|  \| \| \| \| Education \| \| 0 \| \| \| \| . \| \| . \| \| \| \| . \| \| . \| \| \| \| . \| \| \|  \| \| \| \| Post secondary \| \| 1.944 \| \| \| \| 5.594 \| \| 0.35 \| \| \| \| .728 \| \| -9.056 \| \| \| \| 12.944 \| \| \|  \| \| \| \| Asset score \| \| 4.241 \| \| \| \| 1.739 \| \| 2.44 \| \| \| \| .015 \| \| .821 \| \| \| \| 7.662 \| \| \| ** \| \| \| \| Drinks alcohol \| \| 1.302 \| \| \| \| 3.934 \| \| 0.33 \| \| \| \| .741 \| \| -6.434 \| \| \| \| 9.038 \| \| \|  \| \| \| \| Smokes cigarettes \| \| -6.629 \| \| \| \| 3.983 \| \| -1.66 \| \| \| \| .097 \| \| -14.461 \| \| \| \| 1.204 \| \| \| * \| \| \| \| Constant \| \| 73.08 \| \| \| \| 10.919 \| \| 6.69 \| \| \| \| 0 \| \| 51.609 \| \| \| \| 94.551 \| \| \| *** \| \| \| \|  \| \| \| \| \| \| \| \| \| \| \| \| \| \| \| \| \| \| \| \| \| \| \| \| \| Mean dependent var \| \| \| \| 66.988 \| \| \| \| \| SD dependent var \| \| \| \| \| \| \| 44.515 \| \| \| \| \| R-squared \| \| \| \| 0.043 \| \| \| \| \| Number of obs \| \| \| \| \| \| \| 380 \| \| \| \| \| F-test \| \| \| \| 2.862 \| \| \| \| \| Prob > F \| \| \| \| \| \| \| 0.010 \| \| \| \| \| Akaike crit. (AIC) \| \| \| \| 3959.567 \| \| \| \| \| Bayesian crit. (BIC) \| \| \| \| \| \| \| 3987.148 \| \| \| \| \| **** p<.01, ** p<.05, * p<.1* \| \| \| \| \| \| \| \| \| \| \| \| \| \| \| \| \| \| \| \| \| \| \| \| \|  \| \| \| \| \| \| \| \| \| \| \| \| \| \| \| \| \| \| \| \| \| \| \| \|  \| bcfam \| Coef. \| \| St.Err. \| t-value \| \| p-value \| [95% Conf \| \| Interval] \| \| Sig \| \| --- \| --- \| --- \| --- \| --- \| --- \| --- \| --- \| --- \| --- \| --- \| --- \| \| Gender \| 0 \| \| . \| . \| \| . \| . \| \| . \| \|  \| \| Male \| 1.864 \| \| 3.857 \| 0.48 \| \| .629 \| -5.72 \| \| 9.447 \| \|  \| \| Age years \| -.279 \| \| .108 \| -2.58 \| \| .01 \| -.491 \| \| -.066 \| \| ** \| \| Education \| -1.46 \| \| .411 \| -3.55 \| \| 0 \| -2.267 \| \| -.652 \| \| *** \| \| Asset score \| -.372 \| \| .784 \| -0.47 \| \| .635 \| -1.915 \| \| 1.17 \| \|  \| \| Drinks alcohol \| 4.968 \| \| 2.583 \| 1.92 \| \| .055 \| -.112 \| \| 10.047 \| \| * \| \| Smokes cigarettes \| -10.34 \| \| 2.447 \| -4.23 \| \| 0 \| -15.152 \| \| -5.528 \| \| *** \| \| Constant \| 80.764 \| \| 8.167 \| 9.89 \| \| 0 \| 64.705 \| \| 96.822 \| \| *** \| \|  \| \| \| \| \| \| \| \| \| \| \| \| \| Mean dependent var \| \| 47.587 \| \| \| SD dependent var \| \| \| 29.667 \| \| \| R-squared \| \| 0.081 \| \| \| Number of obs \| \| \| 380 \| \| \| F-test \| \| 6.025 \| \| \| Prob > F \| \| \| 0.000 \| \| \| Akaike crit. (AIC) \| \| 3635.584 \| \| \| Bayesian crit. (BIC) \| \| \| 3663.165 \| \| \| **** p<.01, ** p<.05, * p<.1* \| \| \| \| \| \| \| \| \| \| \| \| \|  \| \| \| \| \| \| \| \| \| \| \| \|  \| bcconv \| Coef. \| \| St.Err. \| t-value \| \| p-value \| [95% Conf \| \| Interval] \| \| Sig \| \| --- \| --- \| --- \| --- \| --- \| --- \| --- \| --- \| --- \| --- \| --- \| --- \| \| Gender \| 0 \| \| . \| . \| \| . \| . \| \| . \| \|  \| \| Male \| -36.128 \| \| 41.237 \| -0.88 \| \| .382 \| -117.214 \| \| 44.958 \| \|  \| \| Age years \| -1.215 \| \| 1.118 \| -1.09 \| \| .278 \| -3.413 \| \| .983 \| \|  \| \| Education \| 0 \| \| . \| . \| \| . \| . \| \| . \| \|  \| \| Post secondary \| 4.508 \| \| 40.087 \| 0.11 \| \| .911 \| -74.317 \| \| 83.332 \| \|  \| \| Asset score \| -17.375 \| \| 12.149 \| -1.43 \| \| .154 \| -41.264 \| \| 6.514 \| \|  \| \| Drinks alcohol \| 21.489 \| \| 29.966 \| 0.72 \| \| .474 \| -37.436 \| \| 80.413 \| \|  \| \| Smokes cigarettes \| -24.167 \| \| 30.214 \| -0.80 \| \| .424 \| -83.578 \| \| 35.245 \| \|  \| \| Constant \| 691.524 \| \| 75.175 \| 9.20 \| \| 0 \| 543.704 \| \| 839.345 \| \| *** \| \|  \| \| \| \| \| \| \| \| \| \| \| \| \| Mean dependent var \| \| 558.271 \| \| \| SD dependent var \| \| \| 313.253 \| \| \| R-squared \| \| 0.015 \| \| \| Number of obs \| \| \| 380 \| \| \| F-test \| \| 0.979 \| \| \| Prob > F \| \| \| 0.439 \| \| \| Akaike crit. (AIC) \| \| 5453.493 \| \| \| Bayesian crit. (BIC) \| \| \| 5481.074 \| \| \| **** p<.01, ** p<.05, * p<.1* \| \| \| \| \| \| \| \| \| \| \| \| \|  \| \| \| \| \| \| \| \| \| \| \| \| | | | | | | | | | | | |
| bcprice | Coef. | | St.Err. | t-value | | p-value | [95% Conf | | Interval] | | Sig |
| Gender | 0 | | . | . | | . | . | | . | |  |
| Male | 112.542 | | 76.44 | 1.47 | | .142 | -37.766 | | 262.85 | |  |
| Age years | -.631 | | 2.057 | -0.31 | | .759 | -4.675 | | 3.414 | |  |
| Education | 0 | | . | . | | . | . | | . | |  |
| Post secondary | -88.115 | | 69.421 | -1.27 | | .205 | -224.62 | | 48.391 | |  |
| Asset score | -34.661 | | 20.78 | -1.67 | | .096 | -75.521 | | 6.199 | | * |
| Drinks alcohol | -16.673 | | 50.825 | -0.33 | | .743 | -116.613 | | 83.266 | |  |
| Smokes cigarettes | -104.874 | | 48.648 | -2.16 | | .032 | -200.533 | | -9.215 | | ** |
| Constant | 1162.829 | | 123.369 | 9.43 | | 0 | 920.244 | | 1405.414 | | *** |
|  | | | | | | | | | | | |
| Mean dependent var | | 989.743 | | | SD dependent var | | | 566.308 | |  |  |
| R-squared | | 0.035 | | | Number of obs | | | 380 | |  |  |
| F-test | | 2.027 | | | Prob > F | | | 0.061 | |  |  |
| Akaike crit. (AIC) | | 5895.610 | | | Bayesian crit. (BIC) | | | 5923.191 | |  |  |
| **** p<.01, ** p<.05, * p<.1* | | | | | | | | | | | |
|  | | | | | | | | | | | |

| bcnat | | Coef. | | | | St.Err. | | | t-value | | | p-value | | [95% Conf | | | | Interval] | | | | Sig | |
| --- | --- | --- | --- | --- | --- | --- | --- | --- | --- | --- | --- | --- | --- | --- | --- | --- | --- | --- | --- | --- | --- | --- | --- |
| Gender | | 0 | | | | . | | | . | | | . | | . | | | | . | | | |  | |
| Male | | -9.919 | | | | 24.971 | | | -0.40 | | | .691 | | -59.022 | | | | 39.183 | | | |  | |
| Age years | | .573 | | | | .707 | | | 0.81 | | | .418 | | -.817 | | | | 1.963 | | | |  | |
| Education | | 0 | | | | . | | | . | | | . | | . | | | | . | | | |  | |
| Post secondary | | 6.165 | | | | 23.894 | | | 0.26 | | | .797 | | -40.818 | | | | 53.148 | | | |  | |
| Asset score | | 8.441 | | | | 5.102 | | | 1.65 | | | .099 | | -1.592 | | | | 18.473 | | | |  | |
| Drinks alcohol | | -5.84 | | | | 17.992 | | | -0.32 | | | .746 | | -41.218 | | | | 29.537 | | | |  | |
| Smokes cigarettes | | -15.996 | | | | 18.913 | | | -0.85 | | | .398 | | -53.186 | | | | 21.194 | | | |  | |
| Constant | | 312.554 | | | | 43.462 | | | 7.19 | | | 0 | | 227.092 | | | | 398.016 | | | | *** | |
|  | | | | | | | | | | | | | | | | | | | | | | | |
| Mean dependent var | | | | 308.998 | | | | | | SD dependent var | | | | | | 197.302 | | | | |  |  |  |
| R-squared | | | | 0.015 | | | | | | Number of obs | | | | | | 380 | | | | |  |  |  |
| F-test | | | | 1.006 | | | | | | Prob > F | | | | | | 0.421 | | | | |  |  |  |
| Akaike crit. (AIC) | | | | 5101.872 | | | | | | Bayesian crit. (BIC) | | | | | | 5129.453 | | | | |  |  |  |
| **** p<.01, ** p<.05, * p<.1* | | | | | | | | | | | | | | | | | | | | | | | |
|  | | | | | | | | | | | | | | | | | | | | | | | |
| bcmood | | Coef. | | | | St.Err. | | t-value | | | | p-value | | [95% Conf | | | | Interval] | | | Sig | | |
| Gender | | 0 | | | | . | | . | | | | . | | . | | | | . | | |  | | |
| Male | | -5.722 | | | | 7.029 | | -0.81 | | | | .416 | | -19.543 | | | | 8.098 | | |  | | |
| Age years | | -.392 | | | | .199 | | -1.97 | | | | .05 | | -.783 | | | | 0 | | | ** | | |
| Education | | 0 | | | | . | | . | | | | . | | . | | | | . | | |  | | |
| Post secondary | | 4.793 | | | | 7.152 | | 0.67 | | | | .503 | | -9.27 | | | | 18.856 | | |  | | |
| Asset score | | 4.65 | | | | 1.661 | | 2.80 | | | | .005 | | 1.384 | | | | 7.916 | | | *** | | |
| Drinks alcohol | | 1.975 | | | | 4.983 | | 0.40 | | | | .692 | | -7.825 | | | | 11.774 | | |  | | |
| Smokes cigarettes | | -8.109 | | | | 5.289 | | -1.53 | | | | .126 | | -18.509 | | | | 2.291 | | |  | | |
| Constant | | 125.215 | | | | 12.791 | | 9.79 | | | | 0 | | 100.065 | | | | 150.366 | | | *** | | |
|  | | | | | | | | | | | | | | | | | | | | | | | |
| Mean dependent var | | | | 95.131 | | | | | SD dependent var | | | | | | | 57.437 | | | |  |  |  |  |
| R-squared | | | | 0.065 | | | | | Number of obs | | | | | | | 380 | | | |  |  |  |  |
| F-test | | | | 4.016 | | | | | Prob > F | | | | | | | 0.001 | | | |  |  |  |  |
| Akaike crit. (AIC) | | | | 4144.190 | | | | | Bayesian crit. (BIC) | | | | | | | 4171.771 | | | |  |  |  |  |
| **** p<.01, ** p<.05, * p<.1* | | | | | | | | | | | | | | | | | | | | | | | |
|  | | | | | | | | | | | | | | | | | | | | | | | |

| **Full OLS results food choice motives associated with diet diversity score** |
| --- |

| FVS | Coef. | | St.Err. | t-value | | p-value | [95% Conf | | Interval] | | Sig |
| --- | --- | --- | --- | --- | --- | --- | --- | --- | --- | --- | --- |
| Health | .227 | | .091 | 2.50 | | .013 | .048 | | .406 | | ** |
| Mood | -.041 | | .069 | -0.60 | | .551 | -.177 | | .095 | |  |
| Sensory | .097 | | .057 | 1.71 | | .089 | -.015 | | .209 | | * |
| Weight | -.028 | | .051 | -0.54 | | .588 | -.129 | | .073 | |  |
| Convenience | -.123 | | .058 | -2.13 | | .034 | -.237 | | -.009 | | ** |
| Price | .03 | | .072 | 0.41 | | .679 | -.112 | | .171 | |  |
| Familiarity | .07 | | .049 | 1.44 | | .152 | -.026 | | .165 | |  |
| Natural | -.051 | | .058 | -0.88 | | .379 | -.165 | | .063 | |  |
| Gender | 0 | | . | . | | . | . | | . | |  |
| Male | .038 | | .194 | 0.20 | | .843 | -.343 | | .42 | |  |
| Age years | .002 | | .006 | 0.38 | | .703 | -.01 | | .015 | |  |
| Education | 0 | | . | . | | . | . | | . | |  |
| Beyond secondary school | .49 | | .155 | 3.17 | | .002 | .186 | | .795 | | *** |
| Asset score | .082 | | .05 | 1.64 | | .102 | -.016 | | .18 | |  |
| Smoking status | 0 | | . | . | | . | . | | . | |  |
| Currently smoke | -.147 | | .231 | -0.64 | | .525 | -.6 | | .307 | |  |
| Used to smoke | .112 | | .237 | 0.47 | | .635 | -.353 | | .578 | |  |
| Household size | -.018 | | .046 | -0.38 | | .701 | -.107 | | .072 | |  |
| Gender head HH | 0 | | . | . | | . | . | | . | |  |
| Female | .282 | | .25 | 1.13 | | .259 | -.209 | | .774 | |  |
| What is your marital status | 0 | | . | . | | . | . | | . | |  |
| Currently married | .431 | | .333 | 1.29 | | .197 | -.225 | | 1.086 | |  |
| Separated | .581 | | .365 | 1.59 | | .113 | -.137 | | 1.298 | |  |
| Divorced | .345 | | .534 | 0.65 | | .519 | -.706 | | 1.396 | |  |
| Widowed | .358 | | .356 | 1.01 | | .315 | -.341 | | 1.058 | |  |
| Work type | 0 | | . | . | | . | . | | . | |  |
| Non-government employee | -.195 | | .345 | -0.57 | | .572 | -.873 | | .483 | |  |
| Self-employed | -.181 | | .298 | -0.61 | | .545 | -.767 | | .406 | |  |
| Student | .923 | | .663 | 1.39 | | .165 | -.382 | | 2.228 | |  |
| Homemaker | .004 | | .37 | 0.01 | | .992 | -.724 | | .731 | |  |
| Retired | .084 | | .419 | 0.20 | | .841 | -.74 | | .908 | |  |
| Unemployed (able to work) | .02 | | .367 | 0.06 | | .956 | -.702 | | .743 | |  |
| Unemployed (unable to work) | -.619 | | .413 | -1.50 | | .134 | -1.431 | | .192 | |  |
| Who mainly prepare meals | 0 | | . | . | | . | . | | . | |  |
| House help | -.614 | | .414 | -1.48 | | .139 | -1.428 | | .2 | |  |
| Other female | -.29 | | .228 | -1.27 | | .204 | -.738 | | .158 | |  |
| Father | -.267 | | .31 | -0.86 | | .389 | -.877 | | .343 | |  |
| Other male in the | .09 | | .637 | 0.14 | | .888 | -1.164 | | 1.343 | |  |
| Person making food employed | 0 | | . | . | | . | . | | . | |  |
| yes | .647 | | .163 | 3.97 | | 0 | .326 | | .967 | | *** |
| Drinks alcohol | .097 | | .117 | 0.83 | | .409 | -.133 | | .326 | |  |
| Constant | 6.427 | | .723 | 8.89 | | 0 | 5.005 | | 7.85 | | *** |
|  | | | | | | | | | | | |
| Mean dependent var | | 8.476 | | | SD dependent var | | | 1.342 | |  |  |
| R-squared | | 0.212 | | | Number of obs | | | 380 | |  |  |
| F-test | | 3.743 | | | Prob > F | | | 0.000 | |  |  |
| Akaike crit. (AIC) | | 1278.309 | | | Bayesian crit. (BIC) | | | 1412.274 | |  |  |
| **** p<.01, ** p<.05, * p<.1* | | | | | | | | | | | |
|  | | | | | | | | | | | |

Full ordered logit results food choice motives associated with BMI

**Ordered logistic regression**

| bmi_cat45 | Coef. | | St.Err. | t-value | | p-value | [95% Conf | | Interval] | | Sig |
| --- | --- | --- | --- | --- | --- | --- | --- | --- | --- | --- | --- |
| Health | -.066 | | .153 | -0.43 | | .668 | -.366 | | .235 | |  |
| Mood | -.162 | | .124 | -1.30 | | .192 | -.406 | | .081 | |  |
| Sensory | .579 | | .123 | 4.72 | | 0 | .339 | | .82 | | *** |
| Weight | .192 | | .091 | 2.11 | | .034 | .014 | | .371 | | ** |
| Convenience | -.225 | | .115 | -1.97 | | .049 | -.45 | | -.001 | | ** |
| Price | .054 | | .119 | 0.46 | | .648 | -.178 | | .287 | |  |
| Familiarity | .195 | | .091 | 2.15 | | .032 | .017 | | .373 | | ** |
| Natural | -.229 | | .115 | -1.99 | | .046 | -.454 | | -.004 | | ** |
| Gender | 0 | | . | . | | . | . | | . | |  |
| Male | -1.029 | | .342 | -3.01 | | .003 | -1.699 | | -.36 | | *** |
| Age years | .131 | | .054 | 2.41 | | .016 | .024 | | .237 | | ** |
| Years schooling | .079 | | .037 | 2.12 | | .034 | .006 | | .152 | | ** |
| Asset score | .242 | | .11 | 2.21 | | .027 | .027 | | .457 | | ** |
| wealth2 | -.016 | | .022 | -0.74 | | .459 | -.058 | | .026 | |  |
| Smoking status | 0 | | . | . | | . | . | | . | |  |
| Currently smoke | .392 | | .404 | 0.97 | | .332 | -.4 | | 1.184 | |  |
| Used to smoke | -1.716 | | .428 | -4.01 | | 0 | -2.555 | | -.877 | | *** |
| Household size | -.041 | | .08 | -0.52 | | .605 | -.198 | | .115 | |  |
| Gender head HH | 0 | | . | . | | . | . | | . | |  |
| Female | .098 | | .516 | 0.19 | | .85 | -.914 | | 1.109 | |  |
| What is your marital status | 0 | | . | . | | . | . | | . | |  |
| Currently married | -.052 | | .63 | -0.08 | | .934 | -1.287 | | 1.182 | |  |
| Separated | .459 | | .777 | 0.59 | | .555 | -1.064 | | 1.982 | |  |
| Divorced | -.265 | | 1.024 | -0.26 | | .796 | -2.272 | | 1.743 | |  |
| Widowed | -.244 | | .563 | -0.43 | | .664 | -1.348 | | .859 | |  |
| Work type | 0 | | . | . | | . | . | | . | |  |
| Non-government employee | .685 | | .687 | 1.00 | | .318 | -.661 | | 2.032 | |  |
| Self-employed | .474 | | .601 | 0.79 | | .43 | -.703 | | 1.651 | |  |
| Student | -1.867 | | 1.486 | -1.26 | | .209 | -4.78 | | 1.046 | |  |
| Homemaker | .957 | | .767 | 1.25 | | .212 | -.546 | | 2.461 | |  |
| Retired | .291 | | .845 | 0.34 | | .731 | -1.366 | | 1.948 | |  |
| Unemployed (able to work) | .583 | | .772 | 0.76 | | .45 | -.93 | | 2.096 | |  |
| Unemployed (unable to work) | .578 | | .815 | 0.71 | | .478 | -1.02 | | 2.176 | |  |
| Who mainly prepare meals | 0 | | . | . | | . | . | | . | |  |
| House help | .506 | | .932 | 0.54 | | .587 | -1.321 | | 2.333 | |  |
| Other female | -.105 | | .414 | -0.25 | | .8 | -.918 | | .707 | |  |
| Father | -.463 | | .587 | -0.79 | | .431 | -1.614 | | .688 | |  |
| Other male in the | .108 | | 1.089 | 0.10 | | .921 | -2.026 | | 2.242 | |  |
| Person making food employed | 0 | | . | . | | . | . | | . | |  |
| yes | .748 | | .284 | 2.64 | | .008 | .192 | | 1.304 | | *** |
| Age squared | -.001 | | 0 | -2.12 | | .034 | -.002 | | 0 | | ** |
| Do you exercise | 0 | | . | . | | . | . | | . | |  |
| Yes | -.238 | | .285 | -0.84 | | .402 | -.796 | | .319 | |  |
| Drinks alcohol | -.218 | | .199 | -1.09 | | .275 | -.608 | | .173 | |  |
| cut1 | 2.638 | | 1.868 | .b | | .b | -1.024 | | 6.299 | |  |
| cut2 | 5.675 | | 1.884 | .b | | .b | 1.982 | | 9.368 | |  |
|  | | | | | | | | | | | |
| Mean dependent var | | 2.352 | | | SD dependent var | | | 0.680 | |  |  |
| Pseudo r-squared | | 0.218 | | | Number of obs | | | 369 | |  |  |
| Chi-square | | 156.136 | | | Prob > chi2 | | | 0.000 | |  |  |
| Akaike crit. (AIC) | | 636.215 | | | Bayesian crit. (BIC) | | | 784.826 | |  |  |
| **** p<.01, ** p<.05, * p<.1* | | | | | | | | | | | |
|  | | | | | | | | | | | |
